# Low skeletal muscle mass predicts relevant clinical outcomes in head and neck squamous cell carcinoma. A meta analysis

**DOI:** 10.1177/17588359211008844

**Published:** 2021-05-13

**Authors:** Alexey Surov, Andreas Wienke

**Affiliations:** Department of Radiology and Nuclear Medicine, Otto-von-Guericke-University of Magdeburg, Leipziger Str. 44, Magdeburg, 39112, Germany; Institute of Medical Epidemiology, Biostatistics, and Informatics, Martin-Luther-University Halle-Wittenberg, Halle, Sachsen-Anhalt, Germany

**Keywords:** head and neck cancer, overall survival, sarcopenia

## Abstract

**Background:**

The purpose of this meta-analysis was to analyze the influence of sarcopenia, defined as low skeletal muscle mass, on clinical outcomes in patients with head and neck squamous cell carcinoma (HNSCC) based on a large sample.

**Methods:**

The MEDLINE, EMBASE, and SCOPUS databases were screened for associations between sarcopenia and clinical outcomes in HNSCC up to December 2020. Overall, 27 studies met the inclusion criteria. The methodological quality of the studies involved was checked according to the QUADAS instrument. The meta-analysis was undertaken using RevMan 5.3 software. DerSimonian and Laird random-effects models with inverse-variance weights were used to account for heterogeneity between the studies.

**Results:**

The 27 included studies comprised 7704 patients with different HNSCCs. The cumulative calculated frequency among the studies was 42.0% [95% confidence interval (CI) 35.34–48.65]. Sarcopenia was associated with occurrence of severe postoperative complications, odds ratio (OR) 4.79, 95% CI (2.52–9.11), *p* < 0.00001. Sarcopenia predicted disease-free survival (DFS), simple regression: hazard ratio (HR) 2.00, 95% CI (1.63–2.45), *p* < 0.00001, multiple regression: HR 1.64, 95% CI (1.33–2.03), *p* < 0.00001. Also, sarcopenia was associated with lower overall survival (OS), simple regression: HR 1.96, 95% CI (1.71–2.24), *p* < 0.00001, multiple regression: HR = 1.87, 95% CI (1.53–2.29), *p* < 0.00001. In patients who underwent definitive chemotherapy and/or radiation, sarcopenia predicted lower OS (simple regression), HR 1.95, 95% CI (1.61–2.36), *p* < 0.00001, multiple regression: HR = 1.51, 95% CI (1.17–1.94), *p* < 0.002). In patients with primary surgical strategy with or without adjuvant radio-chemotherapy, sarcopenia was associated with lower OS (simple regression), HR 2.21, 95% CI (1.72–2.84), *p* < 0.00001, multiple regression: HR = 2.05, 95% CI (1.55–2.72), *p* < 0.00001).

**Conclusion:**

The cumulative prevalence of sarcopenia in HNSCC is 42.0%. Sarcopenia is an independent risk factor for OS and DFS in patients with HNSCC who undergo curative therapy. Sarcopenia is associated with the occurrence of severe postoperative complications.

## Introduction

Sarcopenia is a condition defined as a syndrome associated with loss of muscle mass and strength as well as decreased physical performance.^1^ In clinical practice, low skeletal muscle mass (LSMM) on computed tomography (CT) is used as a surrogate marker of sarcopenia.^2–4^ LSMM is a prognostic biomarker predicting disease outcome in different malignancies.^2–7^ So far, it has been shown that sarcopenic patients have higher rates of postoperative major cardiac and/or pulmonary complications in gastric cancer.^2^ In breast cancer, patients with sarcopenia had more grade 3–5 toxicity under chemotherapy compared with non-sarcopenic patients.^3^ In surgically treated non-small cell lung cancer, patients with sarcopenia had a lower 5-year overall survival (OS) rate [risk ratio (RR) = 1.63, 95% confidence interval (CI) = (1.13, 2.33); *p* = 0.008].^4^ In addition, sarcopenia was associated with a lower 5-year disease-free survival (DFS) rate [RR = 1.59, 95% CI = (1.01, 2.52); *p* = 0.046].^4^ Similar results were also reported for pancreatic cancer,^5^ hepatocellular carcinoma,^6^ urothelial carcinoma,^7^ hematological malignancies,^8^ and ovarian cancer.^9^ Loss of skeletal muscle mass during neoadjuvant radiochemotherapy in rectal cancer patients is an independent prognostic factor for DFS and distant metastasis-free survival following curative intent resection.^10^ Some authors indicated that sarcopenia defined as LSMM can also play an essential role also in HNSCC.^11,12^

The purpose of this meta-analysis was to analyze the influence of LSMM on OS in patients with HNSCC based on a large sample.

## Materials and methods

### Data acquisition

**Figure 1.**
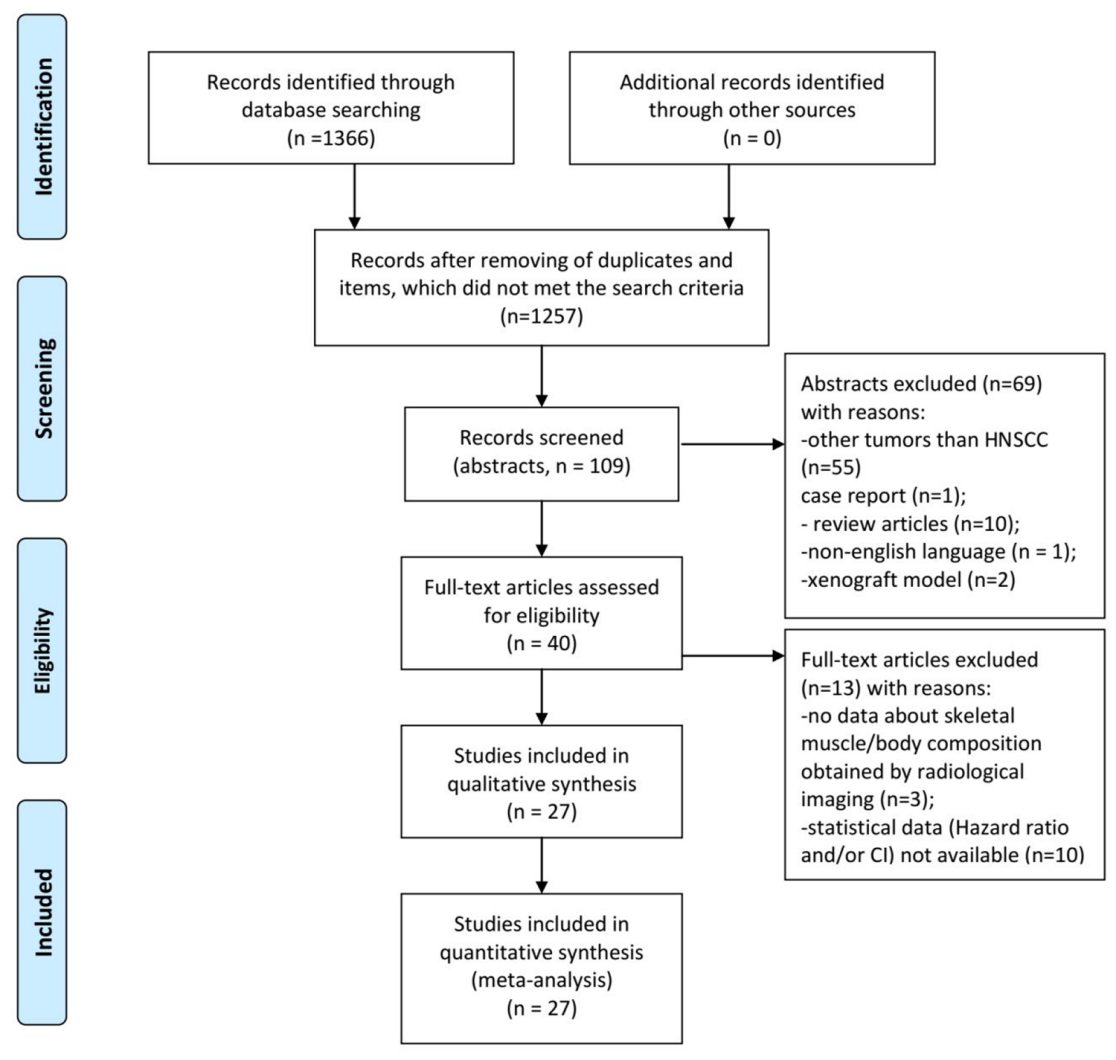
PRISMA flow chart of the data acquisition. HNSCC, head and neck squamous cell carcinoma; PRISMA, preferred reporting items for systematic reviews and meta-analyses.

The MEDLINE library, and Cochrane, EMBASE, and SCOPUS databases were screened for the presence of sarcopenia in HNSCC and associations between LSMM and clinically relevant outcomes like survival, occurrence of complications, and therapy toxicity up to December 2020 (Figure 1).

For data acquisition, the following search criteria were used: “sarcopenia OR low skeletal muscle mass OR body composition AND head neck cancer OR head and neck squamous cell carcinoma OR neck cancer”

The primary search identified 1366 items. Inclusion criteria for the meta analysis were:

- human studies including patients with HNSCC of different origins;
- investigation of pretreatment status of the skeletal musculature by staging computed tomography (CT);
- English language.

Exclusion criteria were:

- Duplicate articles;
- review articles;
- experimental studies used animal models;
- case reports;
- non-English language.

Overall, 1339 articles were excluded and 27 items were included in the analysis. The included 27 articles provided information regarding prevalence of sarcopenia and/or the influence of sarcopenia on complications and survival in patients with HNSCC.^13–39^

The following data were extracted from the included studies: authors, year of publication, diagnosis, number of patients, prevalence of sarcopenia, and statistical data about influence of sarcopenia on clinical outcomes [hazard ratio (HR) and 95% CI]. The Preferred Reporting Items for Systematic Reviews and Meta-Analyses (PRISMA) statement was used for this research.^40^

### Meta-analysis

**Figure 2.**
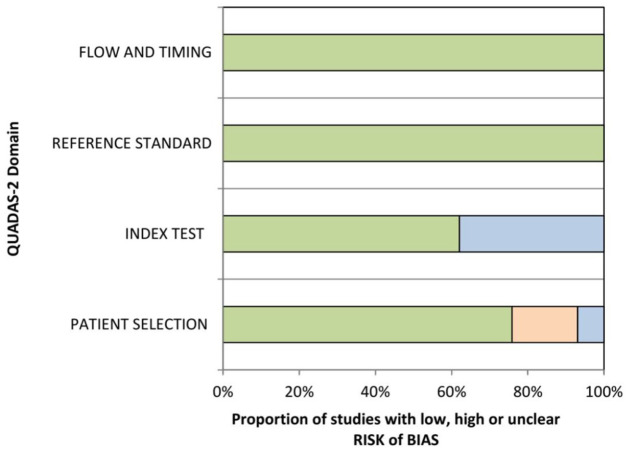
QUADAS-2 quality assessment of the included studies. QUADAS, quality assessment of diagnostic studies.

The methodological quality of the 27 included studies was checked by one observer (AS) using the Quality Assessment of Diagnostic Studies (QUADAS) instrument.^41^ Figure 2 shows the QUADAS results.

The meta-analysis was undertaken using RevMan 5.3 (Computer program, version 5.3. Copenhagen: The Nordic Cochrane Centre, the Cochrane Collaboration, 2014).^42,43^ Heterogeneity was calculated by means of the inconsistency index I^2^. Furthermore, DerSimonian and Laird random-effects models with inverse-variance weights were performed without corrections, as reported previously.^44^

## Results

### Included studies and patients

The 27 studies collected were published predominantly in the years 2019–2020 (*n* = 20, 74%). Most were retrospective (*n* = 24, 89%), with only three studies (11%) of prospective design. The included studies comprised 7704 patients (Table 1). There were 1666 women (21.6%) and 5847 men (75.9%) with a mean age of 62.4 ± 24.8 years. In 191 (2.5%) patients, gender was not reported. The patients had different HNSCC (Table 2). Most frequently, HNSCC of the nasopharynx occurred (*n* = 3633, 47.1%).

**Table 1.** Details of included studies.

Authors	Design	Patients (*n*)	Analyzed clinical values
Achim *et al*.^13^	Retrospective	70	Prevalence
Alwani *et al*.^14^	Retrospective	168	Prevalence, postoperative complications
Ansari *et al*.^15^	Retrospective	78	Prevalence, DFS, OS, postoperative complications
Bril *et al*.^16^	Retrospective	235	Prevalence, postoperative complications, OS
Caburet *et al*.^17^	Retrospective	68	Prevalence
Chargi *et al*.^18^	Retrospective	85	Prevalence, OS
Cho *et al*.^19^	Retrospective	221	Prevalence, OS
Choi *et al*.^20^	Retrospective	79	Prevalence, OS
Fattouh *et al*.^21^	Retrospective	114	OS
Findlay *et al*.^22^	Retrospective	79	Prevalence, OS
Ganju *et al*.^23^	Retrospective	246	Prevalence, OS
Grossberg *et al*.^24^	Retrospective	190	Prevalence, OS
He *et al*.^25^	Prospective	1767	Prevalence, OS
Hua *et al*.^26^	Retrospective	862	Prevalence, OS
Huang *et al*.^27^	Prospective	394	Prevalence
Huiskamp *et al*.^28^	Retrospective	91	Prevalence, DFS, OS
Jung *et al*.^29^	Retrospective	258	Prevalence, DFS, OS
Nakamura *et al*.^30^	Retrospective	106	Prevalence, OS
Nishikawa *et al*.^31^	Retrospective	85	Prevalence
Olson *et al*.^32^	Retrospective	245	Prevalence
Pai *et al*.^33^	Retrospective	881	Prevalence, OS
Schodo *et al*.^34^	Retrospective	41	Prevalence
Stone *et al*.^35^	Retrospective	260	Prevalence, OS
Tamaki *et al*.^36^	Retrospective	113	Prevalence, DFS, OS
van Rijn-Dekker *et al*.^37^	Prospective	744	Prevalence, DFS, OS
Wendrich *et al*.^38^	Retrospective	112	Prevalence
Zwart *et al*.^39^	Retrospective	112	Prevalence

DFS, disease-free survival; OS, overall survival.

**Table 2.** Data regarding patients and tumors.

Patients	*n* (%)
Total	7704
Female	1666 (21.6)
Male	5847 (75.9)
nr	191 (2.5)
Tumor localization	*n* (%)
Oral cavity	463 (6.0)
Nasopharynx	3633 (47.1)
Oropharynx	1555 (20.2)
Hypopharynx	490 (6.4)
Larynx	813 (10.6)
Salivary glands	21 (0.3)
Paranasal sinuses	19 (0.2)
Other (non specified)	710 (9.2)
Tumor stage	*n* (%)
1	302 (3.9)
2	693 (9.0)
3	2092 (27.1)
4	2655 (34.5)
nr	1962 (25.5)

nr, not reported.

In all cases, pretreatment CT images were analyzed for estimation of muscle mass. In most cases (27 studies, 93%), pretreatment skeletal muscle index (SMI) was calculated as a relation: skeletal muscle area divided by the square of the height (cm^2^/m^2^). In detail, in 18 studies (62%), skeletal muscle area was estimated at the third lumbar vertebra. In nine cases (31%), skeletal muscle area was estimated at the third cervical vertebra, and, thereafter, it was converted *via* a special equation to the skeletal muscle area at L3. Different threshold values of SMI were used for the definition of sarcopenia (Table 3). In the remaining two studies (7%), only skeletal muscle areas were estimated.

**Table 3.** Thresholds of LSMM and treatment strategies performed in the included studies.

Authors	Performed treatment	Threshold values for LSMM	
		Men	Women
Achim *et al*.^13^	Surgery alone (total laryngectomy)	52.4 cm^2/m2^	38.5 cm^2/m2^
Alwani *et al*.^14^	Surgery alone	41.6 cm^2/m2^	32.0 cm^2/m2^
Ansari *et al*.^15^	Surgery alone	43.2 cm^2/m2^	43.2 cm^2/m2^
Bril *et al*.^16^	Surgery alone (total laryngectomy)	43.2 cm^2/m2^	43.2 cm^2/m2^
Caburet *et al*.^17^	Surgery alone	52.4 cm^2/m2^	38.5 cm^2/m2^
Chargi *et al*.^18^	Curative treatments, non specified	43.2 cm^2/m2^	43.2 cm^2/m2^
Cho *et al*.^19^	Concurrent CRT or definitive radiotherapy alone	55 cm^2/m2^	39 cm^2/m2^
Choi *et al*.^20^	Definitive RT	605.77 cm^3^	445.42 cm^3^
Fattouh *et al*.^21^	Surgery and CRT	52.4 cm2/m^2^	38.5 cm^2/m2^
Findlay *et al*.^22^	Curative treatments: definitive RT, surgery and adjuvant CRT or RT; definitive CRT	43 cm^2/m2^	41 cm^2/m2^
Ganju *et al*.^23^	Curative treatment: surgery and adjuvant CRT or RT	43 cm^2/m2^	41 cm^2/m2^
Grossberg *et al*.^24^	Curative treatment: definitive RT, surgery and adjuvant CRT or RT; definitive CRT	52.4 cm^2/m2^	38.5 cm^2/m2^
He *et al*.^25^	Definitive RT, surgery and adjuvant CRT or RT; definitive CRT	BMI adjusted^a^	BMI adjusted^a^
Hua *et al*.^26^	Concurrent CRT	18.82 cm^2/m2^	18.82 cm^2/m2^
Huang *et al*.^27^	Concurrent CRT	42.4 cm^2/m2^	42.4 cm^2/m2^
Huiskamp *et al*.^28^	Concomitant cetuximab and RT	45.2 cm^2/m2^	45.2 cm^2/m2^
Jung *et al*.^29^	Definitive treatments: surgery alone; surgery and RT/CRT; RT alone/CRT	52.4 cm^2/m2^	38.5 cm^2/m2^
Nakamura *et al*.^30^	Surgery alone	36.16 cm^2/m2^	31.02 cm^2/m2^
Nishikawa *et al*.^31^	Definitive treatments: surgery alone; RT alone; CRT	46.7 cm^2/m2^	30.3 cm^2/m2^
Olson *et al*.^32^	Definitive treatments: surgery alone; RT alone	52.4 cm^2/m2^	38.5 cm^2/m2^
Pai *et al*.^33^	Definitive treatments: RT alone; CRT	51.74 cm^2/m2^	34.3 cm^2/m2^
Schodo *et al*.^34^	Concurrent CRT	39.7 cm^2/m2^	39.7 cm^2/m2^
Stone *et al*.^35^	Surgery alone	52.4 cm^2/m2^	38.5 cm^2/m2^
Tamaki *et al*.^36^	Curative treatment: definitive RT, surgery and adjuvant CRT or RT; definitive CRT	BMI adjusted^b^	41 cm^2/m2^
van Rijn-Dekker *et al*.^37^	Concurrent CRT or definitive RT alone	42.4 cm^2/m2^	30.6 cm^2/m2^
Wendrich *et al*.^38^	CRT	43.2 cm^2/m2^	43.2 cm^2/m2^
Zwart *et al*.^39^	not reported	43.2 cm^2/m2^	43.2 cm^2/m2^

a For patients with BMI < 30 kg/m^2^, sarcopenia was defined as an SMI of <52 cm^2^/m^2^. For men and <38 cm^2^/m^2^ for women. For patients with BMI ⩾ 30 kg/m^2^, sarcopenia was defined as an SMI of <54 cm^2^/m^2^ for men and <47 cm^2^/m^2^ for women.

b For males, SMI < 43 cm^2^/m^2^ is defined as sarcopenic if the patient is in the BMI category of underweight (<20.0 kg/m^2^) or normal weight (20.0–24.9 kg/m^2^). Overweight (25.0–29.9 kg/m^2^) and obese (>30.0 kg/m^2^) men are considered sarcopenic with an SMI < 41 cm^2^/m^2^. For females, all BMI categories are defined as sarcopenic if SMI is <41 cm^2^/m^2^.

BMI, body mass index; CRT, chemo-radiotherapy; LSMM, low skeletal muscle mass; RT, radiotherapy; SMI, skeletal muscle index.

In most cases (26 studies, 7619 patients) different curative treatments were performed (Table 3). In one study (85 patients), a heterogeneous cohort with both curative and palliative treatment strategies was analyzed.

### Prevalence of sarcopenia

**Figure 3.**
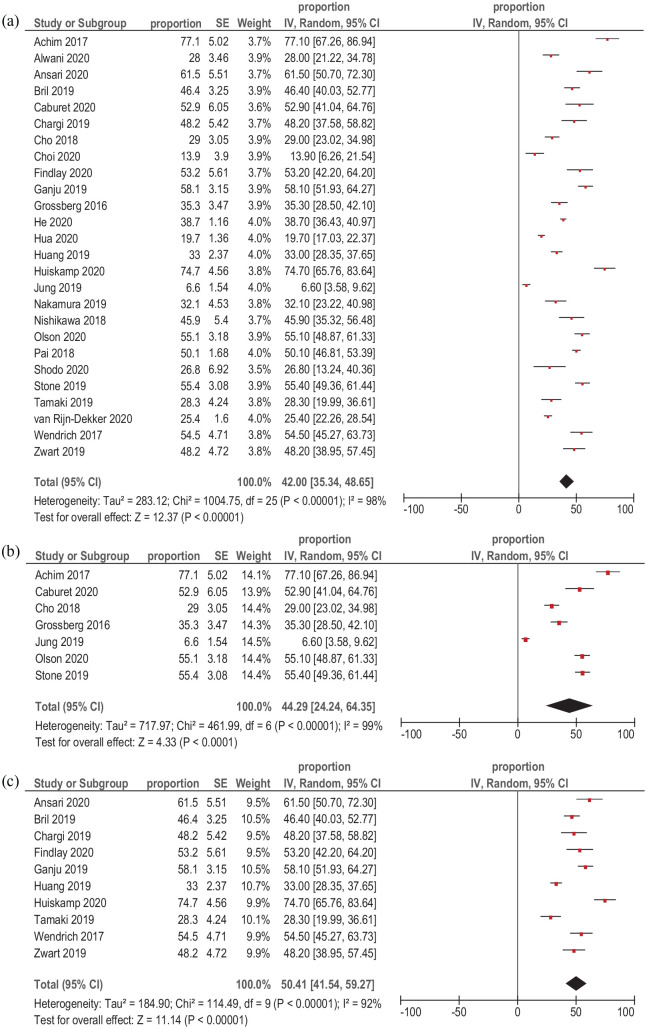
Forest plots of reported prevalences of sarcopenia in patients with HNSCC. (a) Cumulative calculated prevalence among all studies. (b) Cumulative calculated prevalence among studies that used thresholds of 52.4 cm^2^/m^2^ for male patients and 38.5 cm^2^/m^2^ for female patients. (c) Cumulative calculated prevalence among studies that used thresholds of 41.0–45.2 cm^2^/m^2^. CI, confidence interval; HNSCC, head and neck squamous cell carcinoma; SE, standard error.

The prevalence of sarcopenia was reported in 26 studies (7590 patients). It ranged from 6.6% to 77%. The cumulative calculated prevalence among all included studies was 42.0% CI95% (35.34–48.65) (Figure 3a).

At the next step, the prevalence of sarcopenia in dependency on the reported SMI thresholds was calculated. In the subgroups that used thresholds of 52.4 cm^2^/m^2^ for male patients and 38.5 cm^2^/m^2^ for female patients (seven studies, 1312 patients), the cumulative calculated prevalence among the studies was 44.29% I95%C (24.24–64.35) (Figure 3b). In the subgroups that used thresholds of 41.0–45.2 cm^2^/m^2^ for all patients (10 studies, 1545 patients), the cumulative calculated prevalence among the studies was 50.41% 95% CI (41.54–59.27) (Figure 3c).

The remaining studies used different threshold values and, therefore, no other subgroups could be composed.

### Postoperative complications

**Figure 4.**
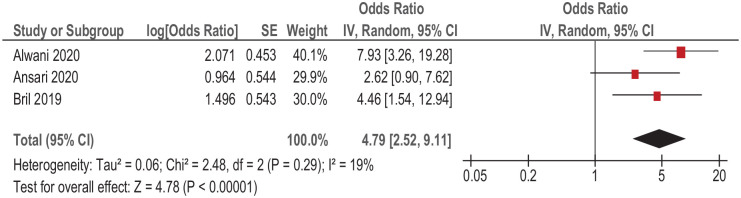
Forest plots of reported HRs of sarcopenia regarding to occurrence of severe postoperative complications (three or more points according to the Clavien–Dindo classification) in patients with HNSCC. CI, confidence interval; HNSCC, head and neck squamous cell carcinoma; HR, hazard ratio; SE, standard error.

For this subanalysis, only reported data on the occurrence of severe complications according to the Clavien–Dindo classification of surgical complications were collected. Associations between the presence of preoperative sarcopenia and occurrence of postoperative complications were analyzed in three studies (481 patients with HNSCC). Simple regression of the collected data showed that sarcopenia was associated with occurrence of severe (three or more points according to the Clavien-Dindo classification) postoperative complications, OR 4.79, 95% CI (2.52–9.11), *p* < 0.00001 (Figure 4). Heterogeneity between the studies was low (*I*^2^ = 19%).

### Disease-free survival

**Figure 5.**
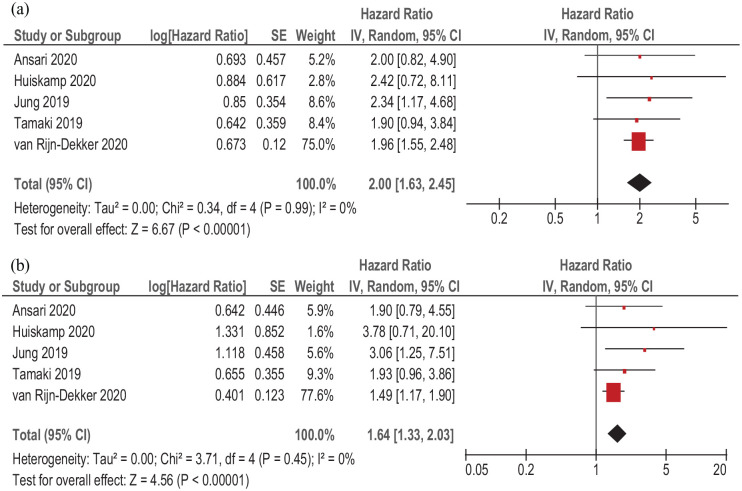
Forest plots of reported HRs of sarcopenia relating to DFS in patients with HNSCC. (a) Unadjusted HRs. (b) Adjusted HRs. CI, confidence interval; DFS, disease-free survival; HNSCC, head and neck squamous cell carcinoma; HR, hazard ratio; SE, standard error.

Associations between sarcopenia and DFS were investigated in five studies (1284 patients). Different curative treatment strategies were performed in the acquired studies. Simple regression of the acquired data showed that sarcopenia predicted DFS in patients with HNSCC, HR 2.00, 95% CI (1.63–2.45), *p* < 0.00001 (Figure 5a). There was no heterogeneity between the included studies (*I*^2^ = 0%).

Also, multiple regression identified that sarcopenia predicted DFS, HR 1.64, 95% CI (1.33–2.03), *p* < 0.00001 (Figure 5b). There was no heterogeneity between the acquired studies (*I*^2^ = 0%).

### Overall survival

**Figure 6.**
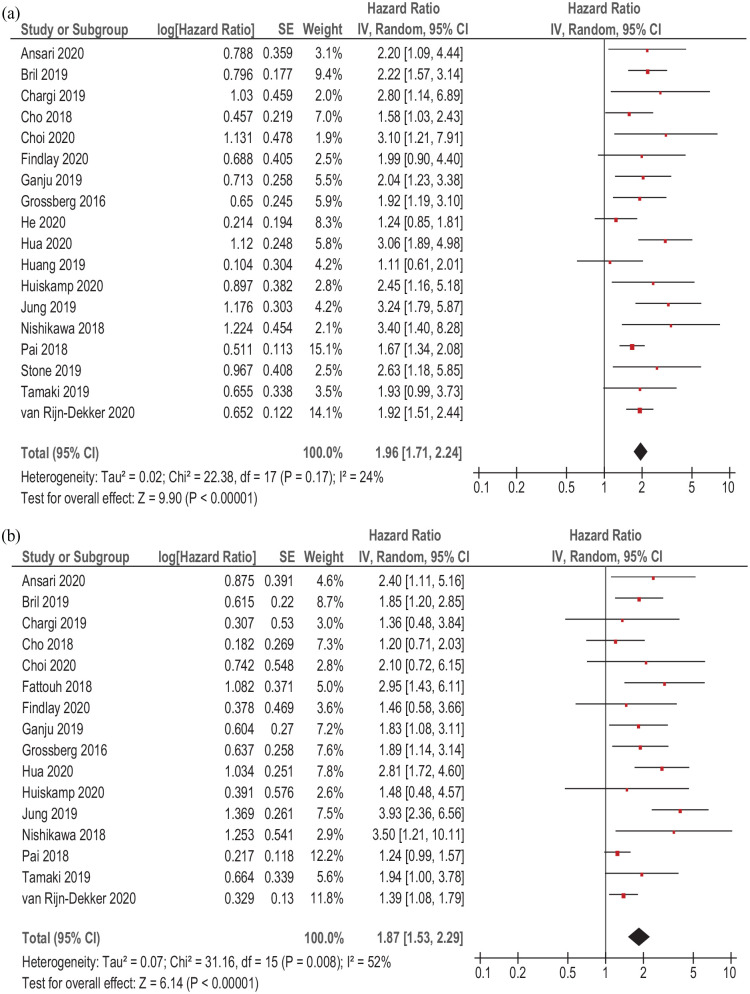
Forest plots of reported HRs of sarcopenia with regard to OS in patients with HNSCC. (a) Unadjusted HRs. (b) Adjusted HRs. CI, confidence interval; HNSCC, head and neck squamous cell carcinoma; HR, hazard ratio; OS, overall survival; SE, standard error.

In 18 studies (6388 patients), relationships between sarcopenia and OS in HNSCC were analyzed. Sarcopenia was associated with lower OS (simple regression), HR 1.96, 95% CI (1.71–2.24), *p* < 0.00001 (Figure 6a). Heterogeneity between the studies was low (*I*^2^ = 24%).

Furthermore, adjusted HRs of sarcopenia were studied. Meta-analysis (multiple regression) identified that adjusted sarcopenia was also associated with lower OS, HR = 1.87, 95% CI (1.53–2.29), *p* < 0.008 (Figure 6b). Heterogeneity among the studies was 52%.

**Figure 7.**
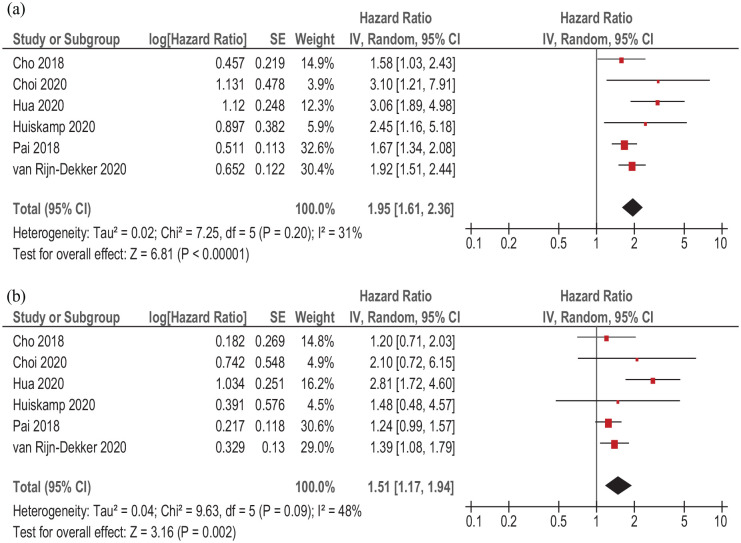
Forest plots of reported HRs of sarcopenia with regard to OS in patients with HNSCC treated by curative radio-chemotherapy. (a) Unadjusted HRs. (b) Adjusted HRs. CI, confidence interval; HNSCC, head and neck squamous cell carcinoma; HR, hazard ratio; OS, overall survival; SE, standard error.

On the next step, associations between pretreatment sarcopenia and OS in dependency on treatment strategy were analyzed. In six studies (2878 patients), definitive chemotherapy and/or radiation was performed. In this subgroup, sarcopenia was associated with lower OS (simple regression), HR 1.95, 95% CI (1.61–2.36), *p* < 0.00001 (Figure 7a). Heterogeneity between the studies was 31%.

Adjusted sarcopenia (multiple regression) was also associated with lower OS, HR = 1.51, 95% CI (1.17–1.94), *p* < 0.002) (Figure 7b). Heterogeneity among the studies was 48%.

**Figure 8.**
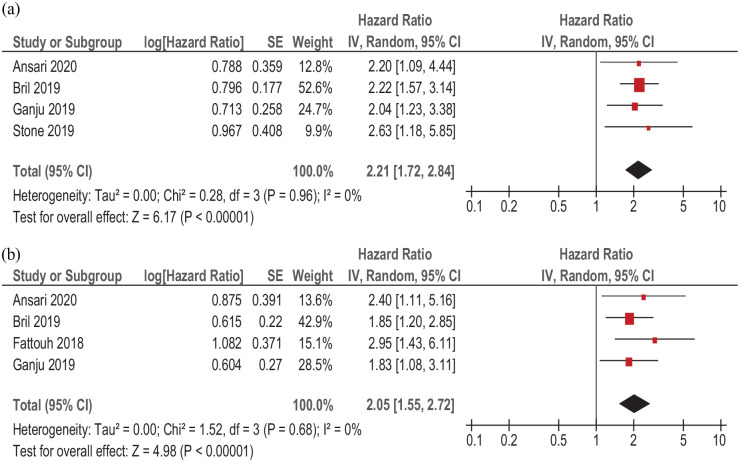
Forest plots of reported HRs of sarcopenia with regard to OS in patients with HNSCC treated by surgery with or without adjuvant radio-chemotherapy. (a) Unadjusted HRs. (b) Adjusted HRs. CI, confidence interval; HNSCC, head and neck squamous cell carcinoma; HR, hazard ratio; OS, overall survival; SE, standard error.

In five studies (933 patients), primary surgical strategy with/or without adjuvant radiochemotherapy was performed. Sarcopenia was associated with lower OS (simple regression), HR 2.21, 95% CI (1.72–2.84), *p* < 0.00001 (Figure 8a). There was no heterogeneity between the studies (*I*^2^ = 0%). Adjusted sarcopenia (multiple regression) was also associated with lower OS, HR = 2.05, CI95% (1.55–2.72), *p* < 0.00001), without heterogeneity (*I*^2^ = 0%) among the studies (Figure 8b).

In the other studies, different treatment strategies were performed. Therefore, no further subgroups in regard to treatment could be composed.

## Discussion

Our data suggest that LSMM plays an important role in patients with HNSCC. Although numerous previous studies have investigated the role of sarcopenia in HNSCC, the data reported are inconsistent. In fact, the true prevalence of sarcopenia in HNSCC is unknown. As shown, prevalence ranges significantly among the reported studies. The present meta-analysis shows that it occurs in 42.0% of patients with HNSCC. This frequency is high and is caused by several factors. Firstly, HNSCC can mechanically impede the intake of nourishment. Secondly, HNSCC can also cause odynophagia and/or dysphagia. Thirdly, frequent alcohol and tobacco abuse in patients with HNSCC provokes malnutrition.

We hypothesize that sarcopenia can also influence short-term postoperative complications in HNSCC. Our results confirm this assumption. As shown, sarcopenia is associated with occurrence of severe (three or more points according to the Clavien–Dindo classification) postoperative complications in patients with HNSCC. Previously, similar results were published for patients with other malignant tumor. So far, in gastric cancer, sarcopenia also predicts postoperative complications.^45^ Also in colorectal cancer, sarcopenia is associated with high risk of postoperative complications.^46^ As mentioned by Xue, sarcopenia might be a marker of a clinically distinct “frailty syndrome” characterized by declines in physiological reserves, which result in an inability to manage acute stressors.^47^

Importantly, our results show that sarcopenia can predict DFS in HNSCC. Interestingly, no heterogeneity among the studies involved was observed. These stable findings covering simple and multiple regression analyses suggest that sarcopenia really predicts DFS in HNSCC and that the calculated HRs are not influenced by study heterogeneity or other factors.

The principle question is, however, whether sarcopenia can predict OS in HNSCC. If so, it can be used as a biomarker in this tumor entity. Some studies have indicated that low skeletal muscle mass also predicted OS in HNSCC.^11,12^ In agreement with these reports, the present meta-analysis based on a large cohort shows that sarcopenia was associated with lower OS. Remarkably, the calculated HRs among the studies do not differ largely. Moreover, also adjusted HRs of sarcopenia are well comparable with those of simple regression. Our data are in agreement with recently published smaller series.^11,12^ Importantly, sarcopenia can be used as a predictor for OS independent of treatment strategy. As shown, LSMM is associated with OS both in the subgroup treated with curative radio-chemotherapy and in the subgroup treated by surgery.

Overall, the present results have high clinical relevance because the fact that sarcopenia is potentially a modifiable factor, and because identification of sarcopenic patients may allow for early interventions to minimize treatment delays and improve outcomes. In fact, it has been shown that a preoperative exercise and nutritional support program can reduce sarcopenia and improve postoperative outcomes in elderly sarcopenic patients with gastric cancer.^48^ Also, in patients with HNSCC, additive nutrition programs can improve clinical outcomes.^49^

Our analysis has some limitations. Firstly, it is based only on results in the English language. Secondly, there are some methodological problems in the included studies; most were retrospective. Some included studies also had high patient selection bias. Thirdly, different approaches were used among the studies to estimate sarcopenia. Most frequently, a measure at the level of L3 from CT images was performed. However, some authors performed a measure at the level of C3 from CT images. Furthermore, we included in the analysis only studies that estimated LSMM on CT. Recently, some reports indicated that ultrasound can also be used successfully to estimate skeletal muscle mass.^50,51^

Fourth, the adjustment variables in the multiple Cox regression models differed in the considered studies. Unfortunately, in all studies, tumors of different origins were pooled and, therefore, no sub-analyses in regard to tumor site and/or stage could be performed. Similarly, no analysis could be performed in regard to tumor grade. Clearly, further studies are needed to overcome the limitations mentioned.

In conclusion, in HNSCC, the cumulative prevalence of sarcopenia defined as LSMM is 42.0%. Sarcopenia is an independent risk factor of OS and DFS in patients with HNSCC who underwent curative therapy. Furthermore, sarcopenia is also associated with occurrence of postoperative complications in HNSCC.

## References

[1] Cruz-Jentoft AJ Bahat G Bauer J, et al. Sarcopenia: revised European consensus on definition and diagnosis. Age Ageing 2019; 48: 16–31. 30312372 10.1093/ageing/afy169 PMC6322506

[2] Kamarajah SK Bundred J Tan BHL. Body composition assessment and sarcopenia in patients with gastric cancer: a systematic review and meta-analysis. Gastric Cancer 2019; 22: 10–22. 30276574 10.1007/s10120-018-0882-2

[3] Aleixo GFP Williams GR Nyrop KA, et al. Muscle composition and outcomes in patients with breast cancer: meta-analysis and systematic review. Breast Cancer Res Treat 2019; 177: 569–579. 31292800 10.1007/s10549-019-05352-3

[4] Deng HY Hou L Zha P, et al. Sarcopenia is an independent unfavorable prognostic factor of non-small cell lung cancer after surgical resection: a comprehensive systematic review and meta-analysis. Eur J Surg Oncol 2019; 45: 728–735. 30348603 10.1016/j.ejso.2018.09.026

[5] Mintziras I Miligkos M Wächter S, et al. Sarcopenia and sarcopenic obesity are significantly associated with poorer overall survival in patients with pancreatic cancer: systematic review and meta-analysis. Int J Surg 2018; 59: 19–26. 30266663 10.1016/j.ijsu.2018.09.014

[6] Chang KV Chen JD Wu WT, et al. Association between loss of skeletal muscle mass and mortality and tumor recurrence in hepatocellular carcinoma: a systematic review and meta-analysis. Liver Cancer 2018; 7: 90–103. 29662836 10.1159/000484950 PMC5892377

[7] Hu X Dou WC Shao YX, et al. The prognostic value of sarcopenia in patients with surgically treated urothelial carcinoma: a systematic review and meta-analysis. Eur J Surg Oncol 2019; 45: 747–754. 30871883 10.1016/j.ejso.2019.03.003

[8] Surov A Wienke A. Sarcopenia predicts overall survival in patients with malignant hematological diseases: a meta-analysis. Clin Nutr 2021; 40: 1155–1160. 32768316 10.1016/j.clnu.2020.07.023

[9] Ubachs J Ziemons J Minis-Rutten IJG, et al. Sarcopenia and ovarian cancer survival: a systematic review and meta-analysis. J Cachexia Sarcopenia Muscle 2019; 10: 1165–1174. 31389674 10.1002/jcsm.12468 PMC6903439

[10] Levolger S van Vledder MG Alberda WJ, et al. Muscle wasting and survival following pre-operative chemoradiotherapy for locally advanced rectal carcinoma. Clin Nutr 2018; 37: 1728–1735. 28756039 10.1016/j.clnu.2017.06.028

[11] Wong A Zhu D Kraus D, et al. Radiologically defined sarcopenia affects survival in head and neck cancer: a meta-analysis. Laryngoscope 2021; 131: 333–341. 32220072 10.1002/lary.28616

[12] Hua X Liu S Liao JF, et al. When the loss costs too much: a systematic review and meta-analysis of sarcopenia in head and neck cancer. Front Oncol 2020; 9: 1561. 10.3389/fonc.2019.01561 PMC7012991 32117787

[13] Achim V Bash J Mowery A, et al. Prognostic indication of sarcopenia for wound complication after total laryngectomy. JAMA Otolaryngol Head Neck Surg 2017; 143: 1159–1165. 28448668 10.1001/jamaoto.2017.0547

[14] Alwani MM Jones AJ Novinger LJ, et al. Impact of sarcopenia on outcomes of autologous head and neck free tissue reconstruction. J Reconstr Microsurg 2020; 36: 369–378. 32088918 10.1055/s-0040-1701696

[15] Ansari E Chargi N van Gemert JTM, et al. Low skeletal muscle mass is a strong predictive factor for surgical complications and a prognostic factor in oral cancer patients undergoing mandibular reconstruction with a free fibula flap. Oral Oncol 2020; 101: 104530. 31881447 10.1016/j.oraloncology.2019.104530

[16] Bril SI Pezier TF Tijink BM, et al. Preoperative low skeletal muscle mass as a risk factor for pharyngocutaneous fistula and decreased overall survival in patients undergoing total laryngectomy. Head Neck 2019; 41: 1745–1755. 30663159 10.1002/hed.25638 PMC6590286

[17] Caburet C Farigon N Mulliez A, et al. Impact of nutritional status at the outset of assessment on postoperative complications in head and neck cancer. Eur Ann Otorhinolaryngol Head Neck Dis. Epub ahead of print 20 December 2019. DOI: 10.1016/j.anorl.2019.12.005. 31870765

[18] Chargi N Bril SI Emmelot-Vonk MH, et al. Sarcopenia is a prognostic factor for overall survival in elderly patients with head-and-neck cancer. Eur Arch Otorhinolaryngol 2019; 276: 1475–1486. 30830300 10.1007/s00405-019-05361-4 PMC6458984

[19] Cho Y Kim JW Keum KC, et al. Prognostic significance of sarcopenia with inflammation in patients with head and neck cancer who underwent definitive chemoradiotherapy. Front Oncol 2018; 8: 457. 30460194 10.3389/fonc.2018.00457 PMC6232888

[20] Choi Y Ahn KJ Jang J, et al. Prognostic value of computed tomography-based volumetric body composition analysis in patients with head and neck cancer: feasibility study. Head Neck 2020; 42: 2614–2625. 32543090 10.1002/hed.26310

[21] Fattouh M Chang GY Ow TJ, et al. Association between pretreatment obesity, sarcopenia, and survival in patients with head and neck cancer. Head Neck 2019; 41: 707–714. 30582237 10.1002/hed.25420 PMC6709588

[22] Findlay M Brown C De Abreu Lourenço R, et al. Sarcopenia and myosteatosis in patients undergoing curative radiotherapy for head and neck cancer: impact on survival, treatment completion, hospital admission and cost. J Hum Nutr Diet 2020; 33: 811–821. 32609428 10.1111/jhn.12788

[23] Ganju RG Morse R Hoover A, et al. The impact of sarcopenia on tolerance of radiation and outcome in patients with head and neck cancer receiving chemoradiation. Radiother Oncol 2019; 137: 117–124. 31085391 10.1016/j.radonc.2019.04.023

[24] Grossberg AJ Chamchod S Fuller CD, et al. Association of body composition with survival and locoregional control of radiotherapy-treated head and neck squamous cell carcinoma. JAMA Oncol 2016; 2: 782–789. 26891703 10.1001/jamaoncol.2015.6339 PMC5080910

[25] He WZ Jiang C Liu LL, et al. Association of body composition with survival and inflammatory responses in patients with non-metastatic nasopharyngeal cancer. Oral Oncol 2020; 108: 104771. 32485608 10.1016/j.oraloncology.2020.104771

[26] Hua X Liao JF Huang X, et al. Sarcopenia is associated with higher toxicity and poor prognosis of nasopharyngeal carcinoma. Ther Adv Med Oncol 2020; 12: 1758835920947612. 10.1177/1758835920947612 PMC7444117 32913446

[27] Huang X Ma J Li L, et al. Severe muscle loss during radical chemoradiotherapy for non-metastatic nasopharyngeal carcinoma predicts poor survival. Cancer Med 2019; 8: 6604–6613. 31517443 10.1002/cam4.2538 PMC6825977

[28] Huiskamp LFJ Chargi N Devriese LA, et al. The predictive and prognostic value of low skeletal muscle mass for dose-limiting toxicity and survival in head and neck cancer patients receiving concomitant cetuximab and radiotherapy. Eur Arch Otorhinolaryngol 2020; 277: 2847–2858. 32335709 10.1007/s00405-020-05972-2 PMC7496017

[29] Jung AR Roh JL Kim JS, et al. Prognostic value of body composition on recurrence and survival of advanced-stage head and neck cancer. Eur J Cancer 2019; 116: 98–106. 31185387 10.1016/j.ejca.2019.05.006

[30] Nakamura H Makiguchi T Yamaguchi T, et al. Impact of sarcopenia on postoperative surgical site infections in patients undergoing flap reconstruction for oral cancer. Int J Oral Maxillofac Surg 2020; 49: 576–581. 31601472 10.1016/j.ijom.2019.09.011

[31] Nishikawa D Hanai N Suzuki H, et al. The impact of skeletal muscle depletion on head and neck squamous cell carcinoma. ORL J Otorhinolaryngol Relat Spec 2018; 80: 1–9. 29393251 10.1159/000485515

[32] Olson B Edwards J Stone L, et al. Association of sarcopenia with oncologic outcomes of primary surgery or definitive radiotherapy among patients with localized oropharyngeal squamous cell carcinoma. JAMA Otolaryngol Head Neck Surg 2020; 146: e201154. 10.1001/jamaoto.2020.1154 PMC7290710 32525518

[33] Pai PC Chuang CC Chuang WC, et al. Pretreatment subcutaneous adipose tissue predicts the outcomes of patients with head and neck cancer receiving definitive radiation and chemoradiation in Taiwan. Cancer Med 2018; 7: 1630–1641. 29608254 10.1002/cam4.1365 PMC5943483

[34] Shodo R Yamazaki K Ueki Y, et al. Sarcopenia predicts a poor treatment outcome in patients with head and neck squamous cell carcinoma receiving concurrent chemoradiotherapy. Eur Arch Otorhinolaryngol. Epub ahead of print 8 August 2020. DOI: 10.1007/s00405-020-06273-4. 32772234

[35] Stone L Olson B Mowery A, et al. Association between sarcopenia and mortality in patients undergoing surgical excision of head and neck cancer. JAMA Otolaryngol Head Neck Surg 2019; 145: 647–654. 31169874 10.1001/jamaoto.2019.1185 PMC6555480

[36] Tamaki A Manzoor NF Babajanian E, et al. Clinical significance of sarcopenia among patients with advanced oropharyngeal cancer. Otolaryngol Head Neck Surg 2019; 160: 480–487. 30105922 10.1177/0194599818793857

[37] van Rijn-Dekker MI van den Bosch L van den Hoek JGM, et al. Impact of sarcopenia on survival and late toxicity in head and neck cancer patients treated with radiotherapy. Radiother Oncol 2020; 147: 103–110. 32251949 10.1016/j.radonc.2020.03.014

[38] Wendrich AW Swartz JE Bril SI, et al. Low skeletal muscle mass is a predictive factor for chemotherapy dose-limiting toxicity in patients with locally advanced head and neck cancer. Oral Oncol 2017; 71: 26–33. 28688687 10.1016/j.oraloncology.2017.05.012

[39] Zwart AT van der Hoorn A van Ooijen PMA, et al. CT-measured skeletal muscle mass used to assess frailty in patients with head and neck cancer. J Cachexia Sarcopenia Muscle 2019; 10: 1060–1069. 31134765 10.1002/jcsm.12443 PMC6818448

[40] Moher D Liberati A Tetzlaff J, et al. Preferred reporting items for systematic reviews and meta-analyses: the PRISMA statement. PLoS Med 2009; 6: e1000097. 10.1371/journal.pmed.1000097 PMC2707599 19621072

[41] Whiting PF Rutjes AW Westwood ME, et al. QUADAS-2: a revised tool for the quality assessment of diagnostic accuracy studies. Ann Intern Med 2011; 155: 529–536. 22007046 10.7326/0003-4819-155-8-201110180-00009

[42] Leeflang MM Deeks JJ Gatsonis C, et al. Systematic reviews of diagnostic test accuracy. Ann Intern Med 2008; 149: 889–897. 19075208 10.7326/0003-4819-149-12-200812160-00008 PMC2956514

[43] Zamora J Abraira V Muriel A, et al. Meta-DiSc: a software for meta-analysis of test accuracy data. BMC Med Res Methodol 2006; 6: 31. 16836745 10.1186/1471-2288-6-31 PMC1552081

[44] DerSimonian R Laird N. Meta-analysis in clinical trials. Control Clin Trials 1986; 7: 177–188. 3802833 10.1016/0197-2456(86)90046-2

[45] Rinninella E Cintoni M Raoul P, et al. Muscle mass, assessed at diagnosis by L3-CT scan as a prognostic marker of clinical outcomes in patients with gastric cancer: a systematic review and meta-analysis. Clin Nutr 2020; 39: 2045–2054. 31718876 10.1016/j.clnu.2019.10.021

[46] van der Kroft G Bours DMJL Janssen-Heijnen DM, et al. Value of sarcopenia assessed by computed tomography for the prediction of postoperative morbidity following oncological colorectal resection: a comparison with the malnutrition screening tool. Clin Nutr ESPEN 2018; 24: 114–119. 29576348 10.1016/j.clnesp.2018.01.003

[47] Xue QL. The frailty syndrome: definition and natural history. Clin Geriatr Med 2011; 27: 1–15. 21093718 10.1016/j.cger.2010.08.009 PMC3028599

[48] Yamamoto K Nagatsuma Y Fukuda Y, et al. Effectiveness of a preoperative exercise and nutritional support program for elderly sarcopenic patients with gastric cancer. Gastric Cancer 2017; 20: 913–918. 28032232 10.1007/s10120-016-0683-4

[49] Kabarriti R Bontempo A Romano M, et al. The impact of dietary regimen compliance on outcomes for HNSCC patients treated with radiation therapy. Support Care Cancer 2018; 26: 3307–3313. 29671062 10.1007/s00520-018-4198-x

[50] Mueller N Murthy S Tainter CR, et al. Can sarcopenia quantified by ultrasound of the rectus femoris muscle predict adverse outcome of surgical intensive care unit patients as well as frailty? A prospective, observational cohort study. Ann Surg 2016; 264: 1116–1124. 26655919 10.1097/SLA.0000000000001546 PMC4907876

[51] Galli A Colombo M Carrara G, et al. Low skeletal muscle mass as predictor of postoperative complications and decreased overall survival in locally advanced head and neck squamous cell carcinoma: the role of ultrasound of rectus femoris muscle. Eur Arch Otorhinolaryngol 2020; 277: 3489–3502. 32535862 10.1007/s00405-020-06123-3

